# ACSS2 enables melanoma cell survival and tumor metastasis by negatively regulating the Hippo pathway

**DOI:** 10.3389/fmolb.2024.1423795

**Published:** 2024-06-03

**Authors:** Baolu Zhang, Qing Zhu, Di Qu, Mao Zhao, Juan Du, Hengxiang Zhang, Hao Wang, Linhan Jiang, Xiuli Yi, Sen Guo, Huina Wang, Yuqi Yang, Weinan Guo

**Affiliations:** Department of Dermatology, Xijing Hospital, Fourth Military Medical University, Xi’an, China

**Keywords:** ACSS2, Hippo pathway, melanoma, metastasis, EMT

## Abstract

**Introduction:**

Acetyl-CoA synthetase 2 (ACSS2), one of the enzymes that catalyze the conversion of acetate to acetyl-CoA, has been proved to be an oncogene in various cancers. However, the function of ACSS2 is still largely a black box in melanoma.

**Methods:**

The ACSS2 expression was detected in melanoma cells and melanocytes at both protein and mRNA levels. Cell viability, apoptosis, migration and invasion were investigated after ACSS2 knockdown. RNA sequencing (RNA-Seq) technology was employed to identify differentially expressed genes caused by ACSS2 knockdown, which were then verified by immunoblotting analysis. Animal experiments were further performed to investigate the influence of ACSS2 on tumor growth and metastasis *in vivo.*

**Results:**

Firstly, we found that ACSS2 was upregulated in most melanoma cell lines compared with melanocytes. In addition, ACSS2 knockdown dramatically suppressed melanoma cell migration and invasion, whereas promoted cell apoptosis in response to endoplasmic reticulum (ER) stress. Furthermore, tumor growth and metastasis were dramatically suppressed by ACSS2 knockdown *in vivo.* RNA-Seq suggested that the Hippo pathway was activated by ACSS2 knockdown, which was forwardly confirmed by Western blotting and rescue experiments. Taken together, we demonstrated that ACSS2 enables melanoma cell survival and tumor metastasis via the regulation of the Hippo pathway.

**Discussion:**

In summary, this study demonstrated that ACSS2 may promote the growth and metastasis of melanoma by negatively regulating the Hippo pathway. Targeting ACSS2 may be a promising target for melanoma treatment.

## 1 Introduction

Melanoma, a life-threatening form of skin cancer originating from melanocytes, has seen a rise in morbidity over the past decade. In 2020, it was estimated that there were 325,000 new melanoma cases and 57,000 related deaths globally (Arnold et al., 2022). Revolutionary treatments, including immune checkpoint inhibitors and targeted therapy with small molecule kinase inhibitors, have significantly altered the treatment landscape for melanoma. However, due to the cancer’s aggressive and diverse characteristics, issues such as treatment non-responsiveness, the development of resistance, and severe immune-related side effects have hindered the full potential of these therapies (Jogerst and Boland, 2022; Lopes et al., 2022). Consequently, investigating new pathogenesis of melanoma is crucial for developing improved therapeutic strategies and enhancing patient outcomes.

Metabolic reprogramming is widely recognized as one of hallmarks of cancers (Hanahan and Weinberg, 2011), playing a pivotal role in melanoma’s development. This reprogramming allows the tumor cells to adapt to the microenvironment, acquire nutrient, and sustain continuous proliferation (Comerford et al., 2014). In recent years, the significance of the acetate metabolic pathway has been highlighted in cancer metabolism, where it is intimately linked to onset, progression, and prognosis of diverse tumors, such as those in the liver, kidney, and pancreas (Sun et al., 2017; Yao et al., 2020; Zhou et al., 2022). Acetyl-CoA synthetase (ACSS) is crucial in this process, converting acetate and coenzyme A into acetyl-CoA. It is present in three forms: ACSS1, ACSS2, and ACSS3, each having unique subcellular localization and functions (Comerford et al., 2014). As a nucleon-cytosolic enzyme, ACSS2 exhibits a strong preference for acetate, positioning it as the core enzyme in acetate metabolism (Moffett et al., 2020). Previous studies indicates that ACSS2 is implicated in numerous biological processes, including lipid metabolism, oxidative phosphorylation, and histone acetylation (Schug et al., 2016), and is significantly associated with development and prognosis of various cancers such as glioblastoma and liver cancer (Sun et al., 2017; Ciraku et al., 2022). Specifically, the upregulation of ACSS2 Ser-267 has been shown to facilitate the growth of glioblastomas through O-GlcNAc transferase activation (Ciraku et al., 2022). Conversely, a negative relationship exists between ACSS2 levels and hepatocellular carcinoma, with the reduction of ACSS2 in liver cancer cells enhancing their invasiveness and mobility (Sun et al., 2017). Notably, glucose-starved mutant BRAF melanoma cells have demonstrated a dependency on both glutamine and acetate for survival (Lakhter et al., 2016). Despite these insights, the contribution of ACSS2 to melanoma metastasis and the mechanisms involved remain largely unexplored.

The Hippo pathway is a crucial regulatory system in tissue development, controlling organ size and playing an essential role in various aspects of tumor progression, such as cancer cell survival, therapy resistance, invasion, and metastasis (Li H. L. et al., 2021). It encompasses a kinase cascade that includes the upstream kinases MST1/2 and LATS1/2, along with the downstream effectors YAP and TAZ (Pan, 2010). This cascade ultimately phosphorylates YAP/TAZ, preventing their entry into the nucleus (Piccolo et al., 2014). The association between melanoma metastasis and the Hippo pathway has been established, with studies showing that increased YAP activity enhances the invasive potential of melanoma cells. This hyperactivity leads to a transformation in cell behavior from proliferative to invasive, driven by elevated expression of genes like AXL, CYR61, and CRIM (Zhang et al., 2020). Yet, the mechanisms controlling the Hippo pathway in melanoma, particularly through cellular metabolism, were far from understood.

This study aimed to elucidate the role of ACSS2 in melanoma progression, with a particular focus on its impact on tumor cell viability and metastatic potential, alongside the mechanisms involved. Initially, it was noted that ACSS2 levels were markedly elevated in melanoma cell lines compared to normal melanocytes. Further analysis revealed that ACSS2 augmented the invasive and migratory capabilities of melanoma cells. Additionally, under TM stimulation, the absence of ACSS2 was found to encourage apoptosis in these cells. Subsequently, preclinical xenograft and lung metastasis models were established to investigate the influence of ACSS2 on melanoma growth and metastasis *in vivo*. What’s more, RNA-Seq analysis and a series of biochemical experiments were performed to explore the mechanism underlying the role of ACSS2 in melanoma.

## 2 Materials and methods

### 2.1 Cell lines and tissues

Human melanoma cell lines A2058, A375, WM793B, WM35, 451Lu, 1205Lu, UACC62 and mouse melanoma cell B16F10 were purchased from American Type Culture Collection (ATCC). Human epidermal melanocyte NHEM and human melanoma cell line UACC257 were granted by Dr. David Schrama in University Hospital Wurzburg, Germany. NHEM was cultured in 254 medium containing human melanocyte growth supplement (S0025, Gibco) and 5% fetal bovine serum (FBS, Procell). UACC62, UACC257, WM35, and 451Lu cell lines were maintained in RPMI1640 medium (Hyclone) supplemented with 10% FBS. A2058, A375 and B16F10 were cultured in DMEM (Hyclone) supplemented with 10% FBS. All cells were incubated under sterile conditions at 37°C and 5% CO2. All cell lines were tested negative for *Mycoplasma* by the Fourth Military Medical University (Xi’an, China).

### 2.2 siRNA interference, shRNA transduction and lentiviral vectors

According to the manufacturer’s instructions, ACSS2 knockdown with two independent siRNAs in A2058 and A375 was performed by using Liposome 3000 transfection reagent kit (L3000-015, Invitrogen). The siRNAs were designed and synthesized by Tsingke (Beijing, China) and primers were listed in Supplementary Table S1. To gain the stable knockdown of ACSS2, cells were transduced with shRNA (short hairpin RNA) lentiviral vectors targeting *ACSS2* with 1 μg/mL polybrene (Tsingke) overnight. After 48 h of lentivirus incubation, the infected melanoma cells were selected using puromycin (10 μg/mL) for 72 h to obtain a single colony, and knockdown efficiency was determined by Western blot. The shACSS2 sequences are also listed in Supplementary Table S1. Lentiviral vectors were purchased from Tsingke (Beijing, China). For ACSS2 overexpression, the plasmid was designed, synthesized and then packaged as a lentivirus in Tsingke (Beijing, China). The subsequent transduction process was almost consistent with lentivirus knockdown.

### 2.3 Quantitative real-time PCR

Following manufacturer’s instructions, total RNA was extracted using Trizol reagent (15596018, Invitrogen). cDNA was synthesized with PrimeScriptTM RT Master Mix kit (RR036A, TaKaRa) and then detected with SYBR Premix Ex TaqTM II kit (RR820A, TaKaRa). Relative RNA expression was normalized to the internal control β-actin and calculated with 2^−ΔΔCT^ method. All primers were as follows: ACSS2—Forward (5′-3′)CGA​GGC​CCT​GCA​GAA​GTG​TC, Reverse (5′-3′)GAG​TCA​CCC​ATG​CCG​AGC​TC; β-actin—Forward (5′-3′)GGC​TAC​AGC​TTC​ACC​ACC​AC, Reverse (5′-3′)TGC​GCT​CAG​GAG​GAG​C.

### 2.4 Western blot analysis

Protein was extracted from cells by protein extraction reagent (EBC-K318-M, Elabscience). Total protein concentrations were determined using a bicinchoninic acid (BCA) kit (TaKaRa). The proteins were resolved by 10% SDS-PAGE (P0012A, Beyotime) and transferred to polyvinylidene fluoride (PVDF) membranes (IPVH00010, Millipore Sigma). Immunoblots were probed with primary antibodies overnight (SA00001-2, Proteintech) at room temperature for 1 h. The blots were visualized using the ECL-Plus reagent (Millipore). The primary antibodies used are listed in Supplementary Table S2.

### 2.5 Immunofluorescence staining analysis

Paraffin-embedded tissue sections were de-paraffinized and rehydrated with graded ethanol dilutions. After antigen retrieval in Tris-EDTA Buffer (10 mM Tris Base, 1 mM EDTA Solution, 0.05% Tween 20, pH 9.0), goat serum was added to block nonspecific binding for 30 min. Then all samples were incubated with primary antibodies (anti-ACSS2, 1:150, Abcam, ab133664; anti-Ki67 1:1000, Abcam, ab15580; cleaved caspase-3, 1:400, #9661S, Cell Signaling Technology). After incubation overnight at 4°C, tissue sections were washed with PBST and then probed with secondary antibodies (Alexa Fluor^®^ 488 labelled Goat Anti-Rabbit IgG H&L 1:200, ab150077, Abcam; Cy3-labelled Goat Anti-Mouse IgG H&L, 1:200, A0521, Beyotime) in dark for 1 h and were stained with an antifade solution containing DAPI. Images were acquired by an inverted confocal laser scanning microscope (Carl Zeiss AG, Oberkochen, Germany).

### 2.6 Immunohistochemical staining analysis

Melanoma tissues were fixed in 10% neutral buffered formalin and paraffin-embedded. Paraffin-embedded melanoma tissues of tumor tissue microarray (TMA, US Biolab Inc. (MME1004I)) or implanted tumor in nude mice were de-paraffinized and rehydrated with graded ethanol dilutions. After antigen retrieval in Tris-EDTA Buffer (introduced above), block nonspecific binding sites with goat serum for 30 min, followed by primary antibodies (anti-ACSS2, 1:150, LSBio, LS-C334743) at 4°C overnight. Then tissue sections were incubated with by horseradish peroxidase-conjugated goat anti-rabbit/mouse IgG (ComWin Biotech) for 30 min at room temperature. The sections were visualized using 3-amino-9-ethylcarbazole (SOLARBIO SCIENCE and TECHNOLOGY), and then were counterstained with haematoxylin and fixed in glycerol. The area and the integrated optical density (IOD) were measured in each image via image Fiji, and IHC scoring of ACSS2 was evaluated by IOD/area value. IHC scoring was assessed independently by two experienced pathologists who did not know the clinical information of the patients.

### 2.7 Flow cytometry analysis of cell apoptosis

24 h after siRNA transfection, A2058 and A375 melanoma cells were treated with ER stress inducer tunicamycin (TM, Abcam, ab120296) for 24 h. For detection of cell apoptosis, cells were collected after experimental treatment and detected by Annexin V-PE/7-AAD Apoptosis detection kit (Multi Sciences, AT104) according to the products’ instructions. All the stained samples were identified in flow cytometer (Beckman Coulter, Miami, United States of America) at least 10,000 events per sample within an hour. The data were analyzed using FlowJo V10.8.1 software. All experiments were performed in triplicate.

### 2.8 Colony formation assay

Adherent cells that transfected by siRNA were collected, and evenly inoculated into six-well plates at same densities (3 × 10^3^ cells per well) for 10 days. Next, the cells were fixed with 4% paraformaldehyde for 20 min and stained with 0.5% crystal violet (Sigma Aldrich, C0775-25G). The density of colonies was calculated by ImageJ. Independent experiments were repeated at least three times.

### 2.9 Cell viability assay

Cell proliferation was monitored with cell counting kit-8 assay (CCK8). A total of 5000 cells per well were inoculated five replicate wells in 96-well plates. After siRNA transfection, cells were cultured respectively for 24, 48, and 72 h at 37°C with 5% CO^2^. Then, 100 μL Cell Count Kit 8 working solution (mixture of 10 μL CCK-8 reagent and 90 μL DMEM) was added per well and incubated at 37°C for 1–1.5 h. Next, a microplate reader (Bio-Rad) was employed to detect the absorbance of each well at 450 nm.

### 2.10 Transwell migration and invasion assay

The migratory or invasive effect of ACSS2 knockdown on A2058 and A375 was evaluated in transwell chamber (Corning, NY, United States of America) with an 8 μm pore size polycarbonate membrane in 24-well plates. For migration assay, the transfected cells were seeded into the upper chamber with serum-free medium (3.5 × 10^4^ cells), and the bottom of the chamber were supplemented with the DMEM medium with 10% FBS. For the invasion assay, the upper chamber was precoated with Matrigel (BD Biosciences), and the subsequent steps were similar to the migration assay. Then the chambers were incubated for 24–48 h. After removal of cells on the upper layer of the membrane, cells adhering to the lower surface cells were fixed with 4% paraformaldehyde and stained with crystal violet. Five randomly selected fields were recorded under an inverted light microscope. The number of migrated or invaded cells was quantified by ImageJ software (NIH).

### 2.11 RNA-seq

Total RNA was extracted from A2058 cells transfected by siRNA and then treated with the Trizol reagent. All RNA samples process and RNA-Seq services were accomplished by Gene *Denovo* Biotechnology (Guangzhou, China). The RNA-seq data used in this study have been deposited in the National Center for Biotechnology Information’s Sequence Read Archive (Sequence Read Archive study accession code PRJNA1067158; https://www.ncbi.nlm.nih.gov/sra/PRJNA1067158).

### 2.12 Mouse xenograft tumor model and tumor lung metastasis model

To investigate the role of ACSS2 in tumor growth *in vivo*, A2058 cells (3.5×10^6^) of stable ACSS2 knockdown were subcutaneously injected into 6-week-old female BALB/c nude mice, which were then randomly divided into experimental and control groups (n = 7). The tumor growth was monitored by measuring length (L) and width (W) twice a week. Tumor size was calculated by the formula: Volume = 1/2 (L × W^2^). When the subcutaneous tumors reached 20 mm^3^ (approximately 3 weeks), the mice were sacrificed, and simultaneously the xenograft tumors were harvested with their weight recorded. To elucidate the influence of ACSS2 knockdown on tumor metastasis *in vivo*, B16F10 cells (2×10^6^) transduced with shRNA were injected into the tail vein of 6-week-old female C57BL/6 mice, which were randomized into three groups in advance (*n* = 6). Mice were sacrificed 9 days post-injection. Then metastasis was evaluated by counting the visible metastatic foci. All of the animal studies were approved by the Animal Care Committee of the Fourth Military Medical University, and conducted in accordance with its regulations for using animals in research.

### 2.13 Statistical analysis

All data were obtained from at least three independent experiments and presented in the form of mean ± standard deviation (SD). Statistical analysis was performed using GraphPad Prism 9.0 software. Unpaired Student’s t-test or one-way ANOVA with Tukey’s HSD test or two-way ANOVA with Sidak test for *post hoc* analysis was used to statistically analyze the significance as appropriate to compare groups. All statistical tests were two-sided, and *p*-value <0.05 was considered statistically significant.

## 3 Results

### 3.1 The expression of ACSS2 is significantly upregulated in melanoma

**FIGURE 1:**
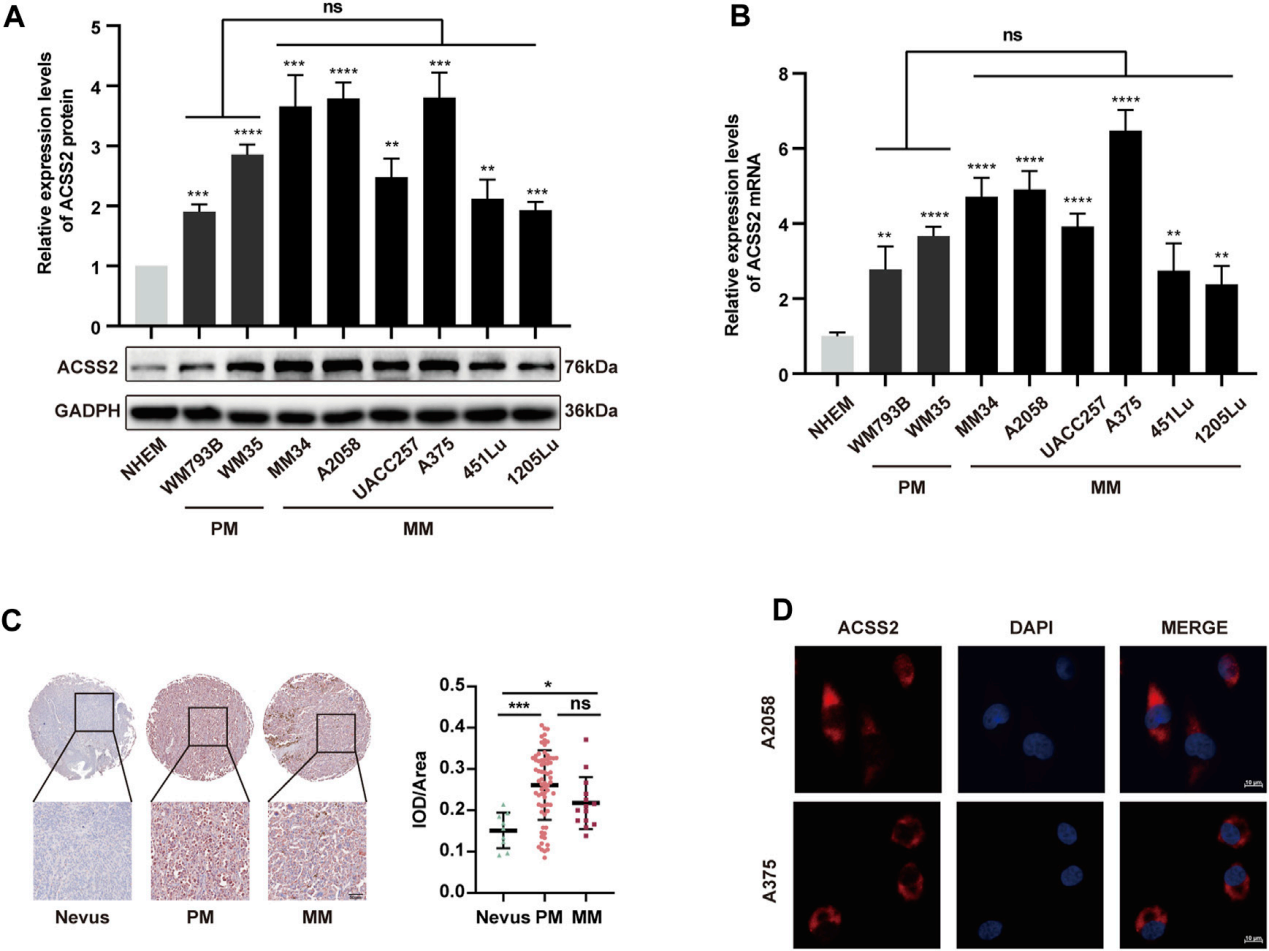
The expression of ACSS2 is significantly upregulated in melanoma. **(A,B)** Relative protein and mRNA levels of ACSS2 in normal human epidermal melanocytes (NHEM) and several melanoma cell lines. **(C)** Immunohistochemical staining analysis of ACSS2 expression in nevus tissues and melanoma tissues. Scale bar = 50 μm. **(D)** Immunofluorescence staining analysis of ACSS2 location in A2058 and A375 melanoma cells. Scale bar = 10 μm. Statistical significance between groups was determined by unpaired Student’s t-test **(A,B)** or one-way ANOVA with Tukey’s HSD test **(C)**. *p < 0.05, **p < 0.01, ***p < 0.001, ****p < 0.0001. NHEM group as control. ns, non-significant. PM, Primary melanoma. MM, Metastatic melanoma.

To explore the association between ACSS2 and melanoma development, we initially assessed the expression of ACSS2 in a panel of eight human melanoma cell lines at different clinical stages (WM793B, WM35 at primary stage and MM34, A2058, UACC257, A375, 451LU, 1205Lu at metastatic stage) and human melanocytes cell line (NHEM). We found that ACSS2 expression was significantly upregulated in melanoma cell lines compared to melanocyte NHEM at both the transcriptional and protein level. Moreover, there was no significant difference in ACSS2 expression between primary and metastatic melanoma cell lines (Figures 1A, B). Furthermore, immunohistochemical analysis was employed to examine ACSS2 expression in TMA and we found that ACSS2 expression was drastically increased in melanoma in comparison with nevus, but was comparable between primary melanoma tissues and metastatic melanoma tissues (Figure 1C). Collectively, these data demonstrate that ACSS2 is upregulated in melanoma. In addition, the immunofluorescence analysis showed that ACSS2 were mainly distributed in the cytoplasm in A2058 and A375 melanoma cells and a small amount can enter the nucleus (Figure 1D), which was consistent with previous studies (Moffett et al., 2020).

### 3.2 ACSS2 promotes melanoma cell survival, invasion and migration

**FIGURE 2:**
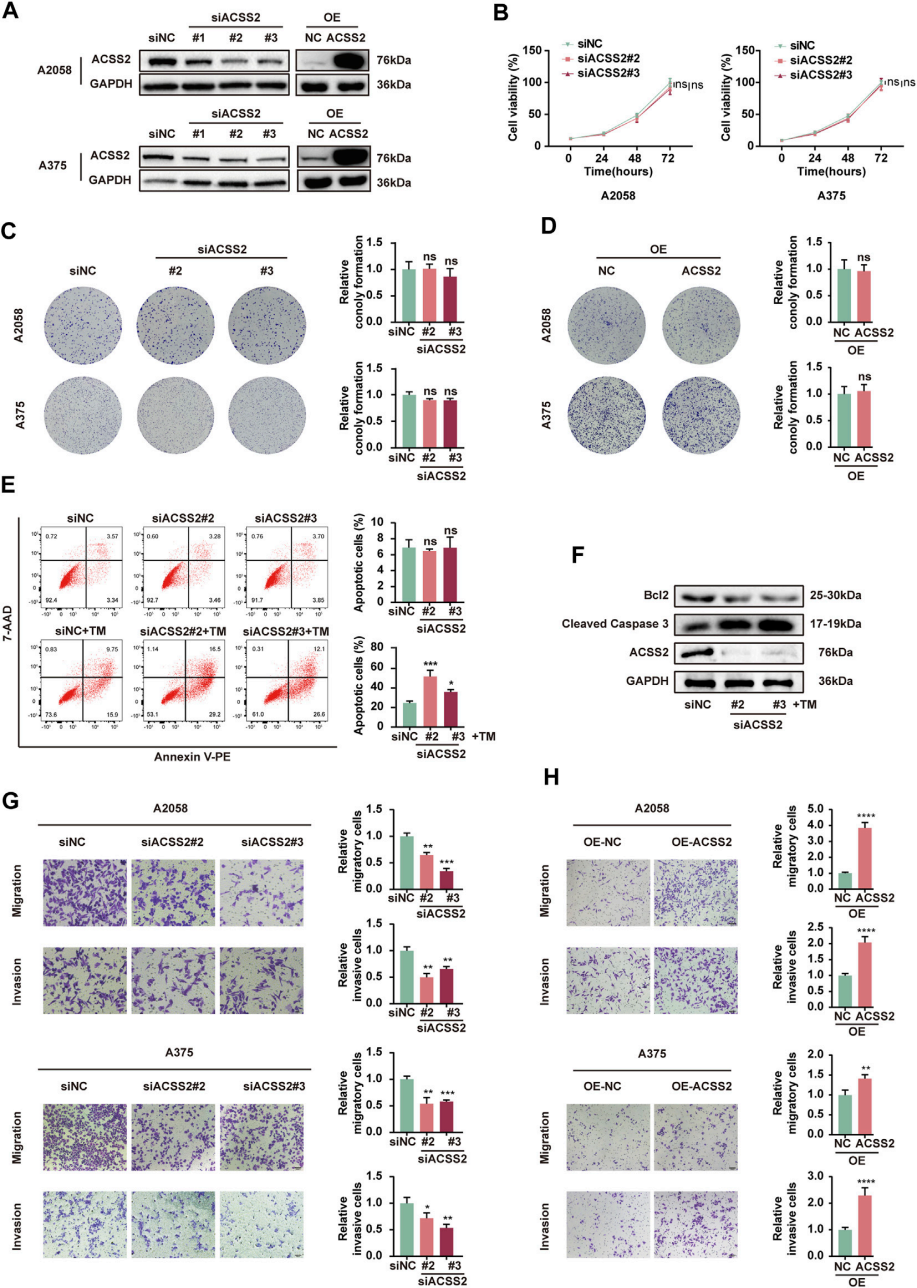
ACSS2 enables melanoma cell survival and promotes melanoma cell invasion and migration. **(A)** The efficiency of knockdown and overexpression of ACSS2 in A2058 and A375 melanoma cells. **(B)** Relative cell viability of A2058 and A375 cells after the knockdown of ACSS2. **(C)** Relative colony formation of both A2058 and A375 cells after the knockdown of ACSS2. **(D)** Relative colony formation of both A2058 and A375 cells after the overexpression of ACSS2. **(E)** Flow cytometry analysis of cell apoptosis in A2058 cells with or without TM treatment (1 μM) after the knockdown of ACSS2. **(F)** Immunoblotting analysis of cell apoptosis in A2058 cells with TM treatment (1 μM) after the knockdown of ACSS2. **(G)** Tumor cell migration and invasion in both A2058 and A375 cells after the knockdown of ACSS2. **(H)** Tumor cell migration and invasion in both A2058 and A375 cells after the overexpression of ACSS2. Statistical significance between groups was determined by two-way ANOVA with Sidak test **(B)** or unpaired Student’s t-test **(C–E,G,H)**. Scale bar = 50 μm *p < 0.05, **p < 0.01, ***p < 0.001, ****p < 0.0001, ns, non-significant.

To investigate the biological role of ACSS2 in melanoma, siRNA transfection was employed to selectively inhibit ACSS2 in A2058 and A375 melanoma cell lines, while its overexpression was achieved through lentiviral vectors in both lines. The efficiency of knockdown and overexpression was validated through Western blot analysis (Figure 2A). The CCK8 assay revealed that melanoma cell viability was not significantly altered by ACSS2 suppression (Figure 2B). Similarly, the colony formation assay indicated that ACSS2 depletion had no significant effect on the clonogenic capacity of melanoma cells (Figure 2C). Moreover, ACSS2 overexpression did not impact colony formation (Figure 2D). Collectively, these findings suggest that ACSS2 is not critical for melanoma cell proliferation under normal conditions.

Then we investigated the effect of ACSS2 on melanoma cells survival. While knockdown of ACSS2 displayed no effect on cell viability under normal condition, it dramatically promoted cell apoptosis under ER stress inducer TM (1 μM) in both melanoma cell lines (Figure 2E, Supplementray Figure S1A). Western blot analysis demonstrated that ACSS2 knockdown increased the expression of pro-apoptotic cleaved caspase-3, but reduced anti-apoptotic Bcl-2 expression after TM treatment (Figure 2F, Supplementray Figure S1B). The phenomenon disclosed that ACSS2 could confer resistance of melanoma cells to apoptosis triggers especially under stressful circumstance. Furthermore, the transwell assay revealed that ACSS2 deficiency significantly reduced the migratory and invasive capabilities of both cell lines, whereas its overexpression could induce the opposite trend. (Figures 2G, H). Taken together, ACSS2 is essential for melanoma cell survival and promotes cell invasion and migration, but is not necessary for cell proliferation under normal conditions.

### 3.3 ACSS2 knockdown suppresses melanoma growth and metastasis *in Vivo*

**FIGURE 3:**
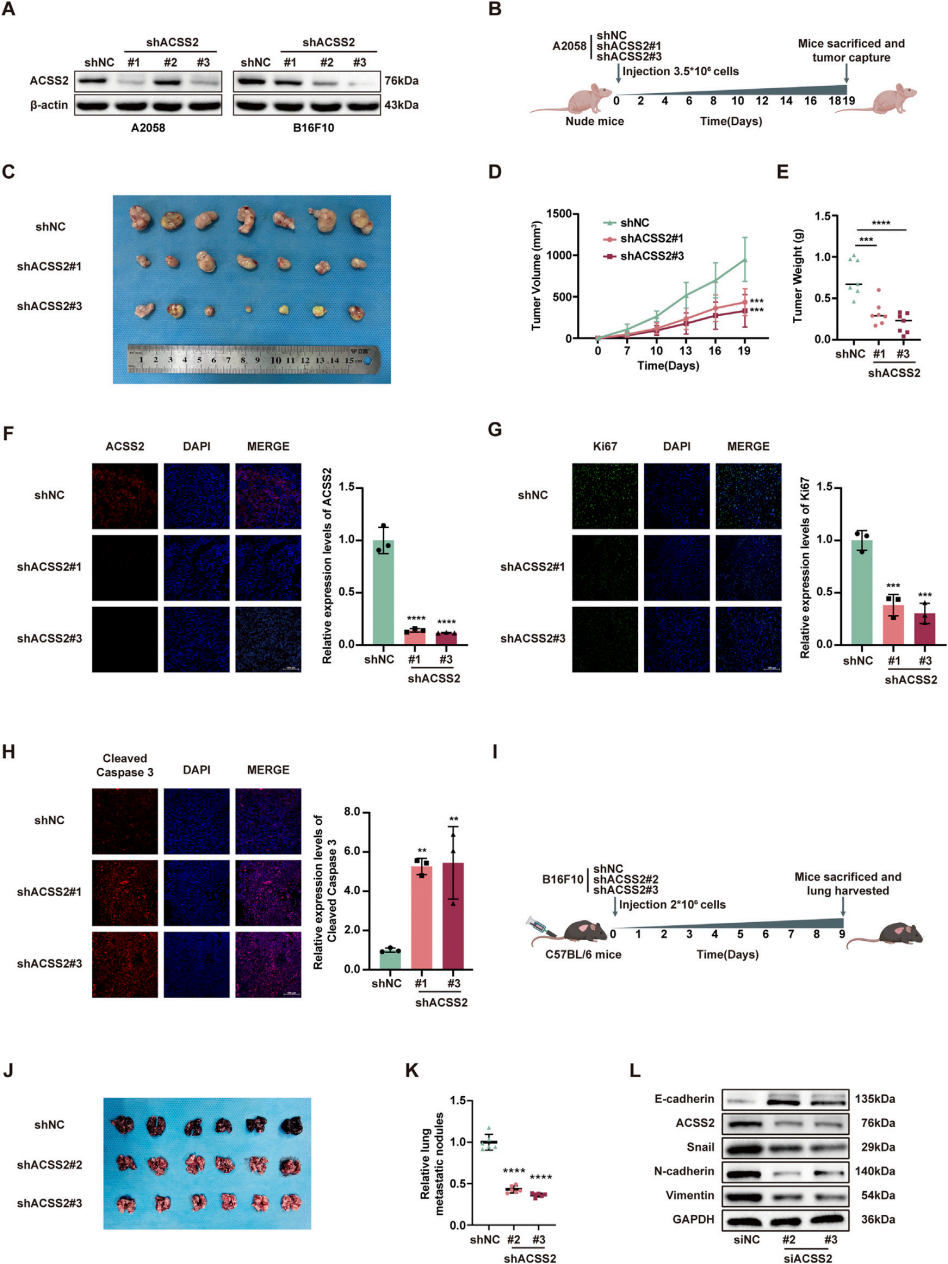
The knockdown of ACSS2 suppresses melanoma progression *in vivo.* **(A)** The efficiency of knockdown of ACSS2 in A2058 and B16F10 melanoma cells. **(B)** Schematic representation of the transplantation plan that BALB/c nude mice burdened with sh-NC or sh-ACSS2#1 or sh-ACSS2#3-transfected A2058 tumors. **(C–E)** Images of A2058 xenografts with indicated transfection isolated from mice. Tumor volumes and weights in each group were calculated and displayed on the right. Symbols of one dot indicates 1 mouse. **(F–H)** Immunofluorescence staining analysis and corresponding fluorescence intensity measurements of ACSS2, Ki-67 and cleaved caspase-3 in isolated tumors with indicated interventions. **(I)** Schematic representation of lung metastasis model via tail vein injection of B16F10 cells into C57BL/6 mice. **(J–K)** Images of lungs isolated from mice in indicated group. The numbers of metastatic nodules in each group were calculated and displayed on the right. Symbols of one dot indicates 1 mouse. **(L)** Immunoblotting analysis of EMT related proteins after the knockdown of ACSS2 in A2058 cells. Statistical significance between groups was determined by two-way ANOVA with Sidak test **(D)** or unpaired Student’s t-test **(E–H,K)**. Scale bar = 100 μm **p < 0.01, ***p < 0.001, ****p < 0.0001. All mouse pictures were created with BioRender.com.

Following the hypothesis that ACSS2 could be a pivotal enhancer of melanoma progression, we established melanoma cell lines with stable ACSS2 knockdown through lentiviral transfection (A2058 and B16F10; Figure 3A). A preclinical xenograft model was then initiated by subcutaneously implanting A2058 cells with ACSS2 knockdown into BALB/c nude mice (Figure 3B). The findings revealed that ACSS2 depletion led to a significant reduction in tumor growth, as evidenced by decreased tumor volume and weight compared to the control group (Figures 3C–E). Moreover, immunofluorescence analysis of the extracted tumors showed an upsurge in the pro-apoptotic marker cleaved-caspase three and a notable decrease in the proliferation marker Ki67 after ACSS2 depletion (Figures 3F–H). Additionally, lung metastasis models were established via the tail vein injection of B16F10 cells with ACSS2 knockdown (Figure 3I). Similarly, the findings indicated a decrease in lung metastases in the group with ACSS2 inhibition compared to the control group (Figures 3J, K). Previous study has shown that epithelial-mesenchymal transition (EMT) is closely related to tumor invasion and metastasis and may be a key step to initiate the invasion and metastasis of malignant tumors (Micalizzi et al., 2010). Given this, we supposed that ACSS2’s role in EMT could be crucial for melanoma’s invasive and metastatic capabilities. Furthermore, immunoblotting analysis to assess EMT marker expression after ACSS2 knockdown in A2058 and A375 lines revealed decreased levels of N-cadherin, Vimentin, and Snail, and increased E-cadherin expression (Figure 3L, Supplementray Figure S1C). These outcomes suggest that ACSS2 may influence melanoma metastasis via EMT regulation. Overall, our findings solidify that ACSS2 promotes melanoma growth and metastasis *in vivo*.

### 3.4 ACSS2 regulates the Hippo pathway in melanoma

**FIGURE 4:**
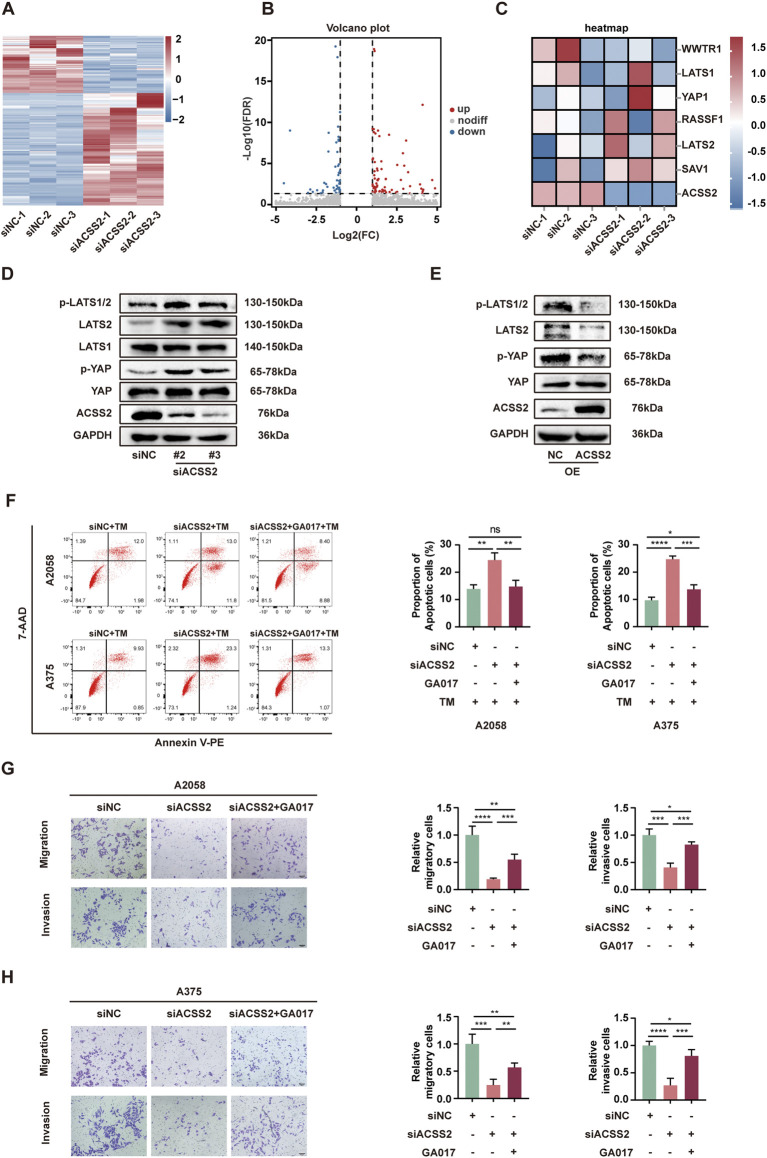
ACSS2 regulates the Hippo pathway in melanoma. **(A)** Heatmap of the differentially expressed genes after ACSS2 knockdown in A2058 cells. **(B)** Volcano plot showing genes with significantly altered expression after ACSS2 knockdown in A2058 cells. Different color indicates different change of expression according to the color legend. **(C)** Heatmap of the genes in the Hippo pathway after ACSS2 knockdown in A2058 cells. **(D)** Immunoblotting analysis of key proteins in the Hippo signaling pathway in A2058 cells after ACSS2 knockdown. **(E)** Immunoblotting analysis of key proteins in the Hippo signaling pathway in A2058 cells after ACSS2 overexpression. **(F)** Flow cytometry analysis of cell apoptosis in A2058 and A375 cells with TM treatment (1 μM) after the knockdown of ACSS2 and pretreated with LATS1/2 inhibitor GA017 (1 μM and 10 μM respectively). **(G)** Tumor cell migration and invasion in A2058 cells after the knockdown of ACSS2 and pretreated with LATS1/2 inhibitor GA017 (1 μM). **(H)** Tumor cell migration and invasion in A375 cells after the knockdown of ACSS2 and pretreated with LATS1/2 inhibitor GA017 (10 μM). Scale bar = 50 μm. Statistical analysis performed by one-way ANOVA with Tukey’s HSD test **(F–H)**. *p < 0.05, **p < 0.01, ***p < 0.001, ****p < 0.0001, ns, non-significant.

To elucidate the mechanism by which ACSS2 knockdown hindered the development of melanoma, we employed RNA-Seq to identify the potential genes regulated by ACSS2. Subsequent analysis demonstrated that 163 genes displayed a more than two-fold expression change in ACSS2-knockdown group compared to controls (Figures 4A, B). Heat map analysis further highlighted that key genes within the Hippo pathway were changed following ACSS2 knockdown (Figure 4C). Subsequent Western blot analysis confirmed that ACSS2 knockdown contributed to the upregulation of LATS2, p-LATS2, and p-YAP, suggesting Hippo pathway activation, while YAP and LATS1 levels remained largely unchanged (Figure 4D, Supplementray Figure S1D). Conversely, these effects were reversed in cells with ACSS2 overexpression (Figure 4E, Supplementray Figure S1E). Given the established connection between the Hippo pathway and melanoma progression (Lamar et al., 2012; Lamar et al., 2019; Thompson, 2020; Zhang et al., 2020; Tan et al., 2021; Zhao et al., 2021), we hypothesized that ACSS2 could promote melanoma progression through the regulation of the Hippo pathway. To verify whether ACSS2’s pro-oncogenic effects in melanoma were mediated via the Hippo pathway, we employed GA-017, a LATS1/2 inhibitor, to deactivate this pathway. Initial tests revealed that GA-017 did not alter ACSS2 expression (Supplementary Figure S1F). Furthermore, treating melanoma cell lines with GA-017 for 72 h after siRNA-induced ACSS2 knockdown partially mitigated the knockdown’s negative effects on cell apoptosis, invasion and migration (Figures 4F–H). Hence, ACSS2’s contribution to melanoma cell survival and metastasis appears to be dependent on the Hippo pathway, underlining the pathway’s critical role in ACSS2-mediated melanoma progression.

## 4 Discussion

Previous studies have elucidated the diverse roles of ACSS2 within oncological contexts, encompassing its participation in lipid synthesis and histone acetylation, as well as its involvement in modulating therapeutic resistance and immune responses within tumors. Additionally, ACSS2 plays a crucial role in tumor metastasis and prognostic assessments. For example, under conditions of nutrient deficiency, acetate serves as the primary carbon source for lipid synthesis. Cytoplasmic ACSS2 catalyzes the conversion of acetate into acetyl-CoA, which subsequently engages in the *de novo* synthesis of fatty acids catalyzed by of FASN (Huang et al., 2018). Under hypoxic conditions, ACSS2 expression is increased in various cancer cell lines including lung, colon, breast, and melanoma, promoting acetate uptake and lipid synthesis, thereby accelerating tumor progression (Yoshii et al., 2009). In hepatocellular carcinoma cells with high ACSS2 expression, acetate enhances the acetylation levels of H3K9, H3K27, and H3K56 in the promoter region through ACSS2, promoting lipid *de novo* synthesis by upregulating ACC and FASN expression, thus enhancing tumor cell survival under hypoxic conditions (Chen et al., 2017). Tamoxifen can induce increased ACSS2 expression in breast cancer cells, leading to treatment resistance, and the combination of ACSS2 inhibitors can reverse this resistance, thereby increasing tamoxifen-induced cell death (Calhoun et al., 2022). Zachary’s research group established that in the context of glucose deprivation, acetate catalyzes histone acetylation in tumor-infiltrating CD8^+^ T cells via an ACSS2-dependent pathway, consequently augmenting IFN-γ production (Qiu et al., 2019). Furthermore, in cervical cancer, ACSS2 expression positively correlates with PD-L1 expression and is closely associated with infiltration by tumor-associated fibroblasts and tumor-associated macrophages (Li C. J. et al., 2021). ACSS2 deletion in murine models of hepatocellular carcinoma resulted in diminished tumor burden, characterized by attenuated tumor proliferation and reduced invasive characteristics (Comerford et al., 2014). Pertaining to renal oncology, an elevation in ACSS2 expression was noted. Inhibition of ACSS2 led to an interruption of the PI3K/AKT signaling cascade, thereby restraining renal carcinoma cell proliferation, migration, and invasion (Zhang et al., 2018). In pancreatic cancer, Li et al. documented that nutritional deficits trigger an upsurge in ACSS2 expression within tumor cells, which in turn stimulates cellular proliferation. ACSS2 enhances pancreatic neoplasia growth through the promotion of H3K27 acetylation at the ETV4 transcription factor promoter region, indirectly activating the ZIP4/CREB pathway and facilitating macropinocytosis. In contrast, ACSS2 knockdown inversely impacts tumor proliferation (Zhou et al., 2022). Furthermore, elevated ACSS2 expression levels have been implicated in adverse prognostic outcomes across various malignancies including hepatic carcinoma, renal carcinoma, and multiple myeloma (Zhang et al., 2018; Wang Y. H. et al., 2019; Li Z. et al., 2021). Similarly, our research has demonstrated that ACSS2 is highly expressed in melanoma cells and contributes to their invasion and migration. Moreover, while ACSS2 minimally influenced melanoma cell viability and colony formation, its suppression notably promoted apoptosis in melanoma cells under ER stress inducer TM. Further investigation confirmed positive role of ACSS2 in facilitating melanoma growth and metastasis *in vivo*. Therefore, we suppose that ACSS2 may promote melanoma growth by regulating cell death rather than cell proliferation.

EMT is a biological process for tumor cells to acquire invasive capacities and it is associated with tumor initiation, invasion and metastasis (Pastushenko and Blanpain, 2019). Previous study revealed that ACSS2 knockdown could reverse the EMT induced by nutrient stress (Su et al., 2019). Notably, in hepatocellular carcinoma, ACSS2 has a negative relationship with HCC malignancy, decreased expression of ACSS2 not only promotes invasion and migration ability of HCC cells, but also promotes EMT (Sun et al., 2017). Likewise, our findings established that ACSS2 knockdown suppressed EMT in melanoma cells. Consequently, ACSS2 may influence cell migration and invasion as well as tumor metastasis in melanoma through the regulation of EMT.

It is well known that the Hippo pathway plays a suppressive role in tumorigenesis. Previous studies have shown that the Hippo pathway is strongly correlated to melanoma cell invasiveness and varied throughout the metastatic cascade in melanoma (Lamar et al., 2012; Lamar et al., 2019; Zhang et al., 2020; Bednarski et al., 2021; Wang et al., 2021; Zhao et al., 2021). Especially the activation of YAP results in uncontrolled melanoma growth and metastasis. A study indicates that triptolide can inhibits aggressive melanoma cell growth and metastasis by inducing SAV1 and LATS1 expression and activation of the Hippo pathway (Tan et al., 2021). Moreover, other studies suggest that there is a connection between YAP/TAZ and EMT in several types of cancer, such as gastric cancer (Wang L. et al., 2019; Nie et al., 2024), pancreatic cancer (Santoro et al., 2018; Ansari et al., 2019; Liu et al., 2023), colorectal cancer (Shao et al., 2014; Ling et al., 2017; Liang et al., 2023) and breast cancer (Lehmann et al., 2016; Feldker et al., 2020). In epithelial cells, the expression of YAP can induce EMT, anchorage-independent growth, aberrant cell proliferation and inhibition of apoptosis (Akrida et al., 2022). Our study indicated that ACSS2 knockdown contributed to the upregulation of LATS2, p-LATS2, and p-YAP, suggesting Hippo pathway activation, while ACSS2 overexpression led to the opposite effect. Furthermore, the administration of GA-017 to melanoma cell lines for 72 h subsequent to ACSS2 knockdown partially reversed the adverse impacts of the knockdown on cell invasion, migration, and apoptosis. Hence, ACSS2’s contribution to melanoma cell invasion, migration and survival and tumor metastasis appears to be dependent on the Hippo pathway. Given the aforementioned context and our experimental results, we identified the Hippo pathway as a plausible intermediary by which ACSS2 governs cell apoptosis and metastasis in melanoma. Additionally, when the Hippo pathway is inactivated in cancer cells, YAP/TAZ enter the nucleus and form complexes with EMT-related transcription factors. YAP/TAZ interact with TEAD transcription factors, enhancing the expression of EMT-related transcription factors and other EMT-related molecules, such as Snail, Slug, and Twist (Akrida et al., 2022). Our research indicates that ACSS2 knockdown can lead to a decrease in Snail. Therefore, we speculate that ACSS2 may promote EMT process by negatively regulating the Hippo pathway, thus promoting melanoma metastasis.

The specific mechanisms by which ACSS2 activates the Hippo pathway have not been fully elucidated. Based on current understanding of ACSS2 function and the regulatory mechanisms of the Hippo pathway, several potential mechanisms could be proposed as follows: 1) The activity of the Hippo pathway is influenced by the cellular metabolic state, especially through energy sensors such as AMPK (Ma et al., 2019). ACSS2 is responsible for converting acetate into acetyl-CoA, which might affect the energy status of cell and impact the activation of AMPK pathway. Therefore, it is of possibility that ACSS2 regulates Hippo pathway and p-LATS2 expression via AMPK. 2) It has been reported that ACSS2 can regulate gene expression by affecting histone acetylation. To be specific, the acetylation state of histones can alter chromatin structure, thereby regulating gene transcription (Li et al., 2017). Therefore, it is also possible that ACSS2 regulates Hippo pathway and p-LATS2 expression by altering histone acetylation at the promoter of specific genes related to the Hippo pathway. In summary, ACSS2 may activate the Hippo pathway through various mechanisms, including influencing cellular metabolism and histone acetylation. The specific mechanisms require further experimental studies for confirmation.

Our study has proven that ACSS2 acts as a tumorigenic factor in melanoma, aggregating tumor growth and metastasis while inhibiting stress-triggered apoptosis, positioning ACSS2 as a viable therapeutic target for melanoma treatment. Currently, inhibitors of ACSS2 have been developed, such as novel substituted tetrazole and amide-substituted condensed pyridine derivatives. These compounds have shown promise in anti-cancer treatment based on initial cell experiments, animal models, and preliminary human safety assessments. However, further research and clinical trials are necessary to confirm their actual effectiveness and safety in cancer treatment (Sabnis, 2021a; Sabnis, 2021b). Taken together, targeting the regulation of acetate metabolism by ACSS2 in melanoma shows promise as a potential therapeutic approach for treating melanoma in future.

## 5 Conclusion

In summary, we demonstrated that ACSS2 knockdown dramatically suppressed melanoma cell migration and invasion, whereas promoted cell apoptosis in response to ER stress. ACSS2 may promote the growth and metastasis of melanoma by negatively regulating the Hippo pathway. Consequently, ACSS2 may serve as a promising target for melanoma treatment.

## Data Availability

The datasets presented in this study can be found in online repositories. The names of the repository/repositories and accession number(s) can be found below: https://www.ncbi.nlm.nih.gov/sra/PRJNA1067158, PRJNA1067158.

## Supplementary material

SUPPLEMENTARY FIGURE S1 ACSS2 promotes melanoma progression via the regulation of the Hippo pathway. **(A)** Flow cytometry analysis of cell apoptosis in A375 cells with or without TM treatment after the knockdown of ACSS2. **(B)** Immunoblotting analysis of cell apoptosis in A375 cells with TM treatment after the knockdown of ACSS2. **(C)** Immunoblotting analysis of EMT related proteins after the knockdown of ACSS2 in A375 cells. **(D)** Immunoblotting analysis of key proteins in the Hippo signaling pathway in A375 cells after ACSS2 knockdown. **(E)** Immunoblotting analysis of key proteins in the Hippo signaling pathway in A375 cells after ACSS2 overexpression. **(F)** Immunoblotting analysis of the expression ACSS2 and LATS2 in A2058 cells and A375 cells pretreated with GA017 at different concentration. Statistical significance between groups was determined by unpaired Student’s t-test. **p* < 0.05, ****p* < 0.001, ns, non-significant.

SUPPLEMENTARY TABLE S1 The sequences of siRNAs and shRNA.

SUPPLEMENTARY TABLE S2 The information of the antibodies used for western blot analysis.
