# Supplementary material for: ACSS2 enables melanoma cell survival and tumor metastasis by negatively regulating the Hippo pathway
Source: Front Mol Biosci. 2024 Jun 3;11:1423795. doi: 10.3389/fmolb.2024.1423795 (PMC11180738; doi:10.3389/fmolb.2024.1423795)
Supplement: Supplementary file 1 [file DataSheet1.docx]

Supplementary Material

# Supplementary Figures and Tables

## Supplementary Figures


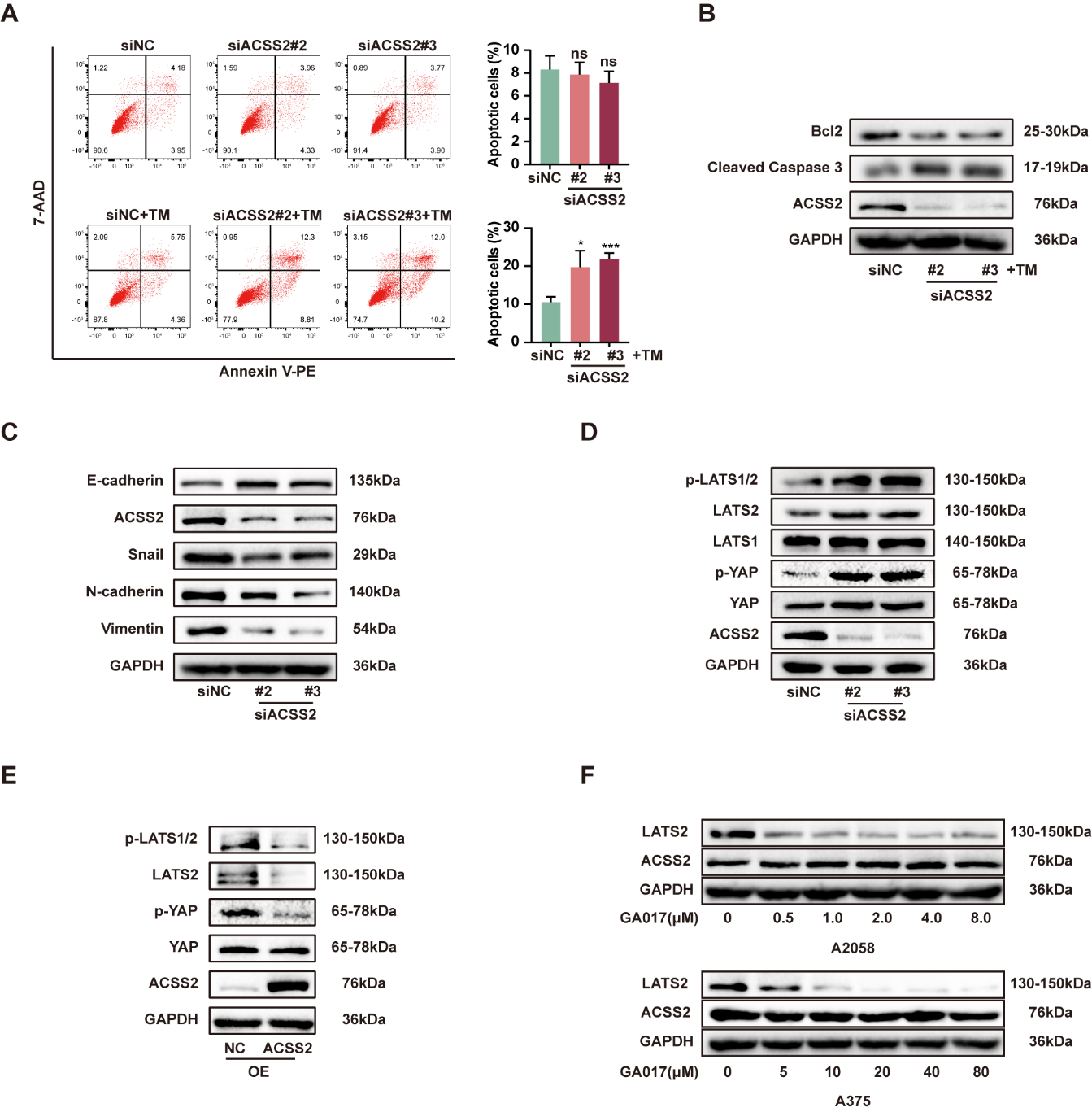


**Figure S1.** **ACSS2 promotes melanoma progression via the regulation of the Hippo pathway.**

(**A**) Flow cytometry analysis of cell apoptosis in A375 cells with or without TM treatment after the knockdown of ACSS2. (**B**) Immunoblotting analysis of cell apoptosis in A375 cells with TM treatment after the knockdown of ACSS2. (**C**) Immunoblotting analysis of EMT related proteins after the knockdown of ACSS2 in A375 cells. (**D**) Immunoblotting analysis of key proteins in the Hippo signaling pathway in A375 cells after ACSS2 knockdown. (**E**) Immunoblotting analysis of key proteins in the Hippo signaling pathway in A375 cells after ACSS2 overexpression. (**F**) Immunoblotting analysis of the expression ACSS2 and LATS2 in A2058 cells and A375 cells pretreated with GA017 at different concentration. Statistical significance between groups was determined by unpaired Student’s t-test. **p* < 0.05, ****p* < 0.001, ns, non-significant.

## Supplementary Tables

### Table S1. The sequences of siRNAs and shRNA

| **Item** | **sense（5'-3'）** | **antisense（5'-3'）** |
| --- | --- | --- |
| si-NC | UUCUCCGAACGUGUCACGUTT | ACGUGACACGUUCGGAGAATT |
| si-ACSS2-1 | GAAGUUUCCUGGAUACUAUTT | AUAGUAUCCAGGAAACUUCTT |
| si-ACSS2-2 | GCUGCAUUGUGGUCAAGCATT | UGCUUGACCACAAUGCAGCTT |
| si-ACSS2-3 | GAGAUGAGCCUGUCACCAATT | UUGGUGACAGGCUCAUCUCTT |
| H-sh-NC | TTCTCCGAACGTGTCACGT | ACGTGACACGTTCGGACAA |
| H-sh-ACSS2-1 | GCTTGGAGATAAAGTTGCTTT | AAAGCAACTTTATCTCCAAGC |
| H-sh-ACSS2-2 | GCTACAATGTACTGGATCGAA | TTCGATCCAGTACATTGTAGC |
| H-sh-ACSS2-3 | CGAACGCTTTGAGACAACCTA | TAGGTTGTCTCAAAGCGTTCG |
| M-sh-NC | GGTTCTCCGAACGTGTCACGT | ACGTGACACGTTCGGAGAACC |
| M-sh-ACSS2-1 | GATCGAAATGTCCATGAGAAA | TTTCTCATGGACATTTCGATC |
| M-sh-ACSS2-2 | GCACACAATTGGAGGCTATAT | ATATAGCCTCCAATTGTGTGC |
| M-sh-ACSS2-3 | GGTTCTGCTTCTTTCCCATTC | GAATGGGAAAGAAGCAGAACC |

H, Human. M, Mouse

### Table S2. The information of the antibodies used for western blot analysis

| **Antibody** | **Usage** | **Product code** |
| --- | --- | --- |
| ACSS2 | 1:1000 | Abcam, ab133664 |
| GAPDH | 1:5000 | Proteintech, 60004-1-Ig |
| β-actin | 1:5000 | Proteintech, 66009-1-Ig |
| LATS1 | 1:1000 | Proteintech, 17049-1-AP |
| LATS2 | 1:1000 | Proteintech, 20276-1-AP |
| p-LATS1/2 | 1:1000 | Affinity Bioscience, AF8163 |
| YAP | 1:1000 | Cell Signalling Technology, #14074 |
| p-YAP | 1:1000 | Cell Signalling Technology, #13008 |
| Bcl2 | 1:1000 | Proteintech, 12789-1-AP |
| Cleaved Caspase 3 | 1:1000 | Cell Signalling Technology, #9664 |
